# Class II obese and healthy pregnant controls exhibit indistinguishable pro‐ and anti‐inflammatory immune responses to Caesarian section

**DOI:** 10.1002/iid3.174

**Published:** 2017-05-25

**Authors:** Caroline Graham, Mullein Thorleifson, William P. Stefura, Duane J. Funk, Kent T. HayGlass

**Affiliations:** ^1^ Department of Immunology University of Manitoba Winnipeg Canada; ^2^ Department of Anesthesia and Perioperative Medicine University of Manitoba Winnipeg Canada

**Keywords:** Caesarian section, cytokines, inflammation, innate immunity, obesity

## Abstract

**Introduction:**

Obesity during pregnancy is associated with meta‐inflammation and an increased likelihood of clinical complications. Surgery results in intense, acute inflammatory responses in any individual. Because obese individuals exhibit constitutive inflammatory responses and high rates of Caesarian section, it is important to understand the impact of surgery in such populations. Whether more pronounced pro‐inflammatory cytokine responses and/or counterbalancing changes in anti‐inflammatory immune modulators occurs is unknown. Here we investigated innate immune capacity in vivo and in vitro in non‐obese, term‐pregnant controls versus healthy, term‐pregnant obese women (Class II, BMI 35–40).

**Methods:**

Systemic in vivo induction of eleven pro‐ and anti‐inflammatory biomarkers and acute phase proteins was assessed in plasma immediately prior to and again following Caesarian section surgery. Independently, innate immune capacity was examined by stimulating freshly isolated PBMC in vitro with a panel of defined PRR‐ligands for TLR4, TLR8, TLR3, and RLR 24 h post‐surgery.

**Results:**

The kinetics and magnitude of the in vivo inflammatory responses examined were indistinguishable in the two populations across the broad range of biomarkers examined, despite the fact that obese women had higher baseline inflammatory status. Deliberate in vitro stimulation with a range of PRR ligands also elicited pro‐ and anti‐inflammatory cytokine responses that were indistinguishable between control and obese mothers.

**Conclusions:**

Acute in vivo innate immune responses to C‐section, as well as subsequent in vitro stimulation with a panel of microbial mimics, are not detectably altered in Class II obese women. The data argue that while Class II obesity is undesirable, it has minimal impact on the in vivo inflammatory response, or innate immunomodulatory capacity, in women selecting C‐section.

## Introduction

Obesity is a global epidemic affecting more than 1.9 billion adults 1. Increased societal obesity is a massive burden on health care systems and society due to its direct and indirect costs 2, 3. Its impacts now exceed those of smoking as the most expensive preventable disorder 3.

Obesity during pregnancy is associated with increased meta‐inflammation 4, 5, 6, 7, 8. It is one of the most common obstetrical risk factors for adverse outcomes 9. It is linked to increased risk of gestational diabetes, placental inflammation, preeclampsia, miscarriage, preterm birth, caesarian section, and stillbirth 4, 6, 7, 10, 11. The clinical complications arising from maternal obesity are dose‐responsive and associate directly with increasing BMI 6. Thus, preeclampsia, the leading cause of maternal and perinatal morbidity, exhibits a stepwise correlation with increasing BMI class 10.

The impact of obesity in pregnancy is not limited to maternal health. Increasing evidence indicates influences of maternal physiology on outcomes in their children that range from development of a healthy microbiome 12 to increased risks of metabolic dysfunction, heart disease, and other chronic inflammatory conditions 13, 14. Taken together, the increasing frequency and intensity of obesity in women of child bearing age is of great concern 9, 15.

Surgery results in multiple intra‐ and post‐operative alterations in all individuals. These include innate immune activation and rapid systemic increases in circulating inflammatory biomarkers that result from the combined impact of tissue damage, anesthesia, postoperative pain, and psychological stress 16, 17, 18. In some individuals, surgery leads to intense systemic inflammatory response syndrome and can result in multi‐organ failure 17. The specific contributions of obesity to normal inflammatory processes, as well its impact on subsequent clinical outcomes including infection, wound healing, embolism, and mortality are areas of active investigation 19.

Whether Caesarian section elicits more pronounced inflammatory responses in obese women, and/or any counterbalancing changes in anti‐inflammatory responses, is unknown. Given that obesity increases the likelihood of women receiving planned or emergent C‐sections 6, it is important to better understand the impact of surgery on pregnant women. Here we test the supposition that pregnant women of BMI 35–40 at term develop exaggerated net inflammatory responses to C‐section. Healthy, term‐pregnant, obese women were compared with non‐obese, term‐pregnant controls to examine in vivo and in vitro innate immune capacity. In vivo responses were examined as changes in systemic induction of pro and anti‐inflammatory biomarkers and acute phase proteins prior to and during the acute surgically induced (C‐section) innate response. Independently, innate immune capacity following surgery was assessed by acutely stimulating fresh PBMC, obtained 24 h after surgery, with a panel of defined PRR‐ligands and comparing pro‐ and anti‐inflammatory cytokine responses. The results demonstrate that while surgery elicits intense acute responses in vivo, their magnitude is indistinguishable in the two populations of women, despite the fact that the obese women exhibit higher baseline inflammatory marker expression pre‐C‐section. Similarly, in vitro stimulation of innate cytokine responses revealed no differences. The results argue that while obesity is clearly to be minimized or avoided, the acute systemic innate immune response to C‐section surgery, or to subsequent stimulation with microbial mimics, is not detectably different in Class II obese women.

## Materials and Methods

### Participants

**Figure 1:**
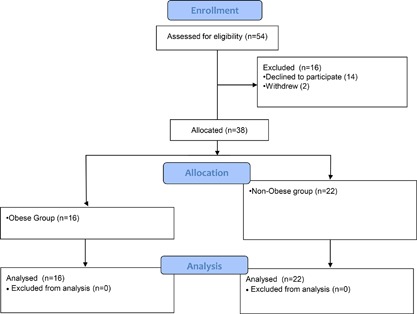
CONSORT diagram.

Following ethics approval from University of Manitoba Institutional Review Board and written informed consent from each participant, 36 healthy pregnant women were enrolled within a day of elective caesarean section (Fig. 1). Sixteen were Class II obese 20 (mean BMI at recruitment 37.7 ± 2.7), and 22 were healthy pregnant controls (mean BMI 28.4 ± 1.7).

Table 1 provides key clinical characteristics of the participants. Inclusion criteria included: Uneventful term pregnancy without documented concerns about hypertension, proteinuria, or gestational diabetes resulting in a healthy baby. One woman had twins. Exclusion criteria included labor at time of C‐section, comorbid inflammatory disease, other chronic diseases (e.g., osteoarthritis or asthma) or current infection. All denied any recent illness. Heparinized blood was drawn at IV start, then again 1, 3, 5, and 24 h post‐surgery in the recovery room. Plasma was collected by centrifugation (500*g*, 10 min), aliquoted and stored at −20°C.

**Table 1:** Clinical description of study participants

Patient number	Normal/obese	BMI	Age	Ethnicity	Indication for OR
1	Normal	27.7	25	Caucasian	Placenta Previa
2	Normal	30.0	35	Filipino	Repeat Elective
3	Normal	28.0	31	Caucasian	Repeat Elective
4	Normal	29.3	30	Caucasian	Repeat Elective
5	Normal	28.7	32	Egyptian	Repeat Elective
6	Normal	30.0	36	Filipino	Repeat Elective
7	Normal	30.0	31	Caucasian	Repeat Elective
8	Normal	30.0	32	Caucasian	Repeat Elective
9	Normal	29.0	33	Caucasian	Repeat Elective
10	Normal	30.2	33	Caucasian	Repeat Elective
11	Normal	25.5	32	Caucasian	Repeat Elective
12	Normal	29.7	32	African Canadian	Repeat Elective
13	Normal	26.9	33	Caucasian	Repeat Elective
14	Normal	28.0	36	Caucasian	Repeat Elective
15	Normal	27.8	36	Chinese	Repeat Elective
16	Normal	28.2	38	Filipino	Repeat Elective
17	Normal	25.6	28	Caucasian	Vasa Previa
18	Normal	25.4	35	Filipino	Repeat Elective
19	Normal	28.2	28	Caucasian	Repeat Elective
20	Normal	30.0	25	African Canadian	Repeat Elective
21	Normal	26.0	27	Filipino	Repeat Elective
22	Normal	30.4	41	Caucasian	Elective Primary
23	Obese	40.7	35	Filipino	Twin Breech
24	Obese	39.0	23	Caucasian	Repeat Elective
25	Obese	35.9	34	Ashkenazi Jewish	Repeat Elective
26	Obese	42.9	28	African Canadian	Breech
27	Obese	34.7	26	Caucasian	Fetal Diaphragmatic Hernia
28	Obese	38.7	29	Caucasian	Breech
29	Obese	34.9	26	Caucasian	Breech
30	Obese	38.6	26	Afghani	Repeat Elective
31	Obese	41.2	37	Caucasian	Repeat Elective
32	Obese	35.5	35	Filipino	Repeat Elective
33	Obese	35.1	28	Caucasian	Repeat Elective
34	Obese	35.4	28	Caucasian	Repeat Elective
35	Obese	40.5	36	Caucasian	Repeat Elective
36	Obese	35.1	33	Caucasian	Repeat Elective
37	Obese	39.7	33	Caucasian	Repeat Elective
38	Obese	35.3	40	Filipino	Repeat Elective

### In vitro culture

Innate stimuli (PRR ligands) representative of bacterial and viral stimuli were used for short term in vitro stimulation of fresh PBMC directly ex vivo. Briefly, PBMC obtained 24 h post‐partum were isolated using Ficoll (GE Healthcare, Mississauga, Canada) gradients, cultured in triplicate at 350,000 cells/well with 200 μL medium alone and in the presence of a TLR4 ligand (0.4 ng/mL LPS, InvivoGen, San Diego, CA), TLR8 ligand (400 ng/mL CL075, InvivoGen), TLR3 ligand (50 µg/mL poly(I:C), InvivoGen) or RLR ligand (100 ng/mL poly(I:C)/Lyovec, InvivoGen). Supernatants were harvested after 24 h culture and stored at −20°C.

### Immunological analyses

Pre‐ and post‐operative samples for individuals were assayed as pairs to control for inter‐assay variation. Meso Scale Discovery (MSD, Rockville, MD) singleplex assays were used to quantify CRP, CCL2, CCL8, CXCL10, TNFα, IL‐6, IL‐10, IL‐1Ra, and sTNF‐RII following manufacturer's protocols. Leptin and PTX3 (R&D Systems, Minneapolis, MN) were quantified with ultrasensitive ELISA protocols as described 21, 22. The concentrations reported are based on standard curves created using serial dilutions of fresh aliquots of constant recombinant lab standards in medium stored at −80°C in individual 400 µL aliquots (Cedarlane, Burlington, Canada; PeproTech, Quebec, Canada; R&D Systems). The median assay coefficient of variation was typically below 5% for MSD assays and between 5% and 15% for ELISA.

### Statistics

Each point represents the average value obtained from duplicate or triplicate analyses of an individual woman's plasma or culture supernatant at that time. Intragroup data were analyzed using pairwise comparisons of medians (Wilcoxon Matched Pairs/Signed Rank tests, GraphPad Prism, La Jolla, CA). Comparisons of obese versus control populations utilized Mann–Whitney analysis. Differences were considered significant at the 95% confidence level (two‐tailed *p* < 0.05).

## Results

### Systemic innate immune status in vivo prior to C‐section

**Figure 2:**
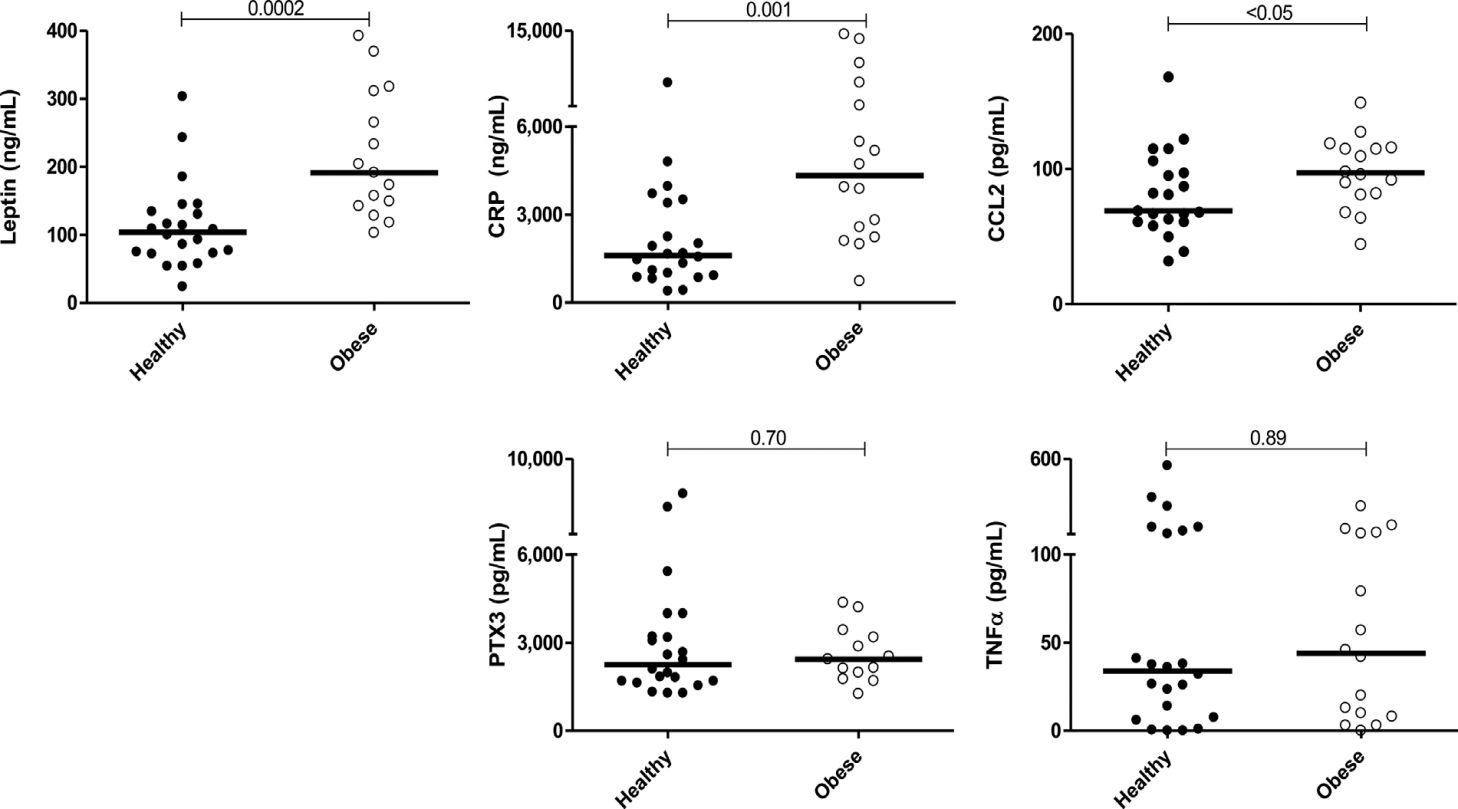
Innate immune biomarker expression just prior to C‐section delivery: Obese term pregnant women express higher basal levels of systemic inflammatory biomarkers than do healthy pregnant controls. Plasma from 22 healthy term pregnant women (mean BMI 28, black circles) was compared with that of 16 Class II obese, term pregnant women (mean BMI 38, open circles). Mann–Whitney *p*‐values shown.

In this longitudinal study, two populations of healthy pregnant women who gave birth to healthy full term infants by elective C‐section after uneventful pregnancies were studied. Initially, to determine the extent to which pregnant women if BMI 35–40 at term exhibited baseline elevations in inflammatory biomarkers, plasma samples were assessed for leptin and other biomarkers characteristic of meta‐inflammation. Figure 2 demonstrates that pregnant women of BMI 35–40 have higher baseline leptin and CRP levels at term than do healthy pregnant controls (*p* = 0.0002, 0.001, respectively). Pentraxin‐3, an early biomarker of acute inflammatory response 23 was indistinguishable between groups (*p* = 0.70). Among systemic innate cytokines and chemokines, median CCL2 was ∼40% elevated in obese women prior to surgery, whereas plasma TNFα was indistinguishable.

### Kinetics of biomarker expression following elective C‐section

**Figure 3:**
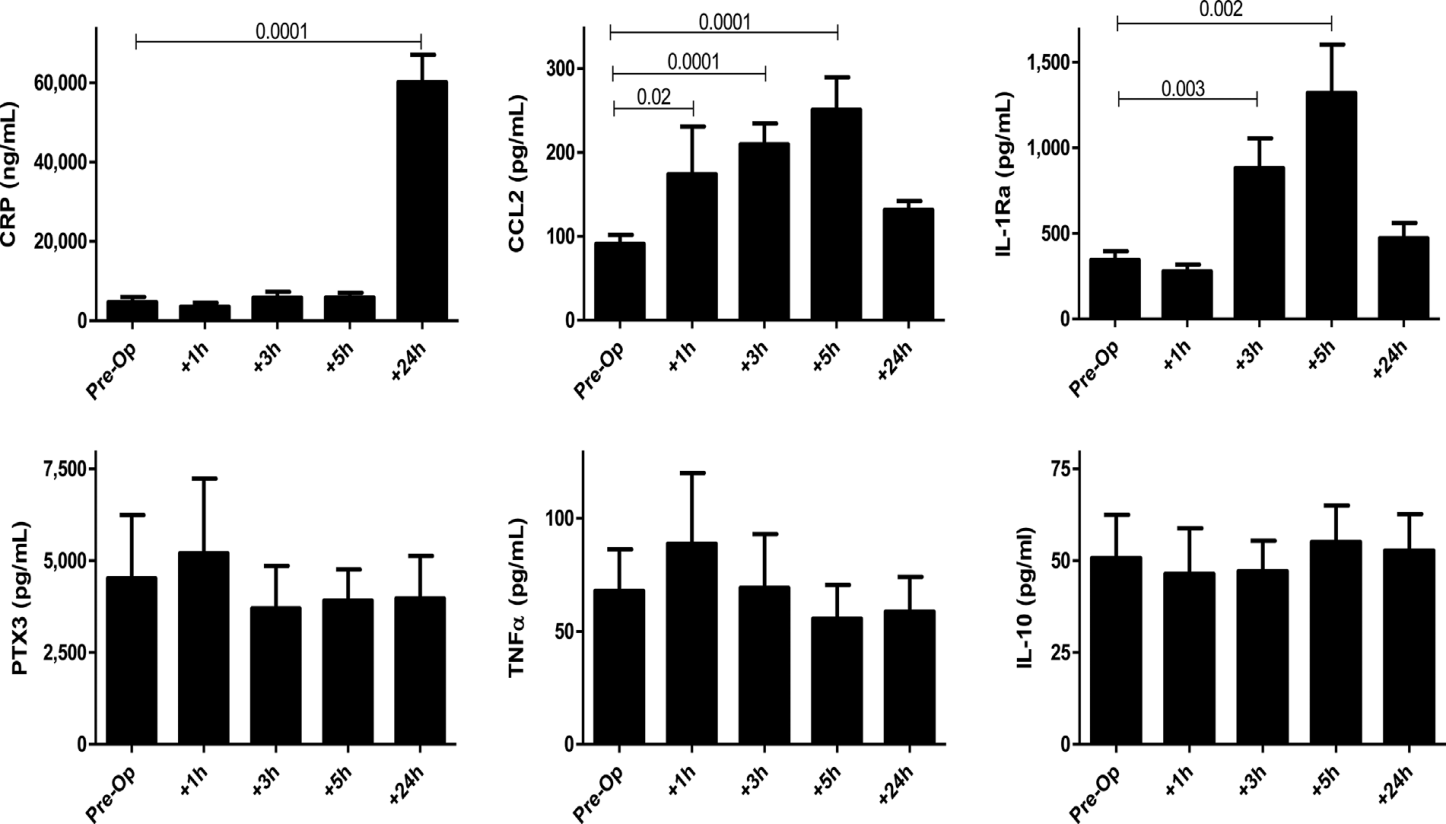
Kinetics of elective Caesarian section stimulated pro and anti‐inflammatory responses in vivo. Mann–Whitney *p*‐values are provided from analysis of ten randomly selected women examined.

Different innate stimuli (i.e., surgery, trauma, infection, immunization) elicit very different kinetics of expression for innate immune cytokine responses. To define (1) which biomarkers were increased upon surgery; and (2) the optimal time points to examine such responses, five pre‐/post‐operative time points were examined. Kinetics of pro‐ and anti‐inflammatory responses following C‐section are shown in Figure 3. For those biomarkers that did demonstrate responses, increases were substantial, ranging from 3 to 13‐fold. Several other biomarkers that were readily found in plasma pre‐surgery, did not exhibit increased expression following C‐section (i.e., PTX3, TNFa, IL‐10, Fig. 3) as well as IL‐6 and sTNF‐RII (data not shown).

### Pro‐inflammatory, anti‐inflammatory, and acute phase protein responses in obese women post‐surgery

**Figure 4:**
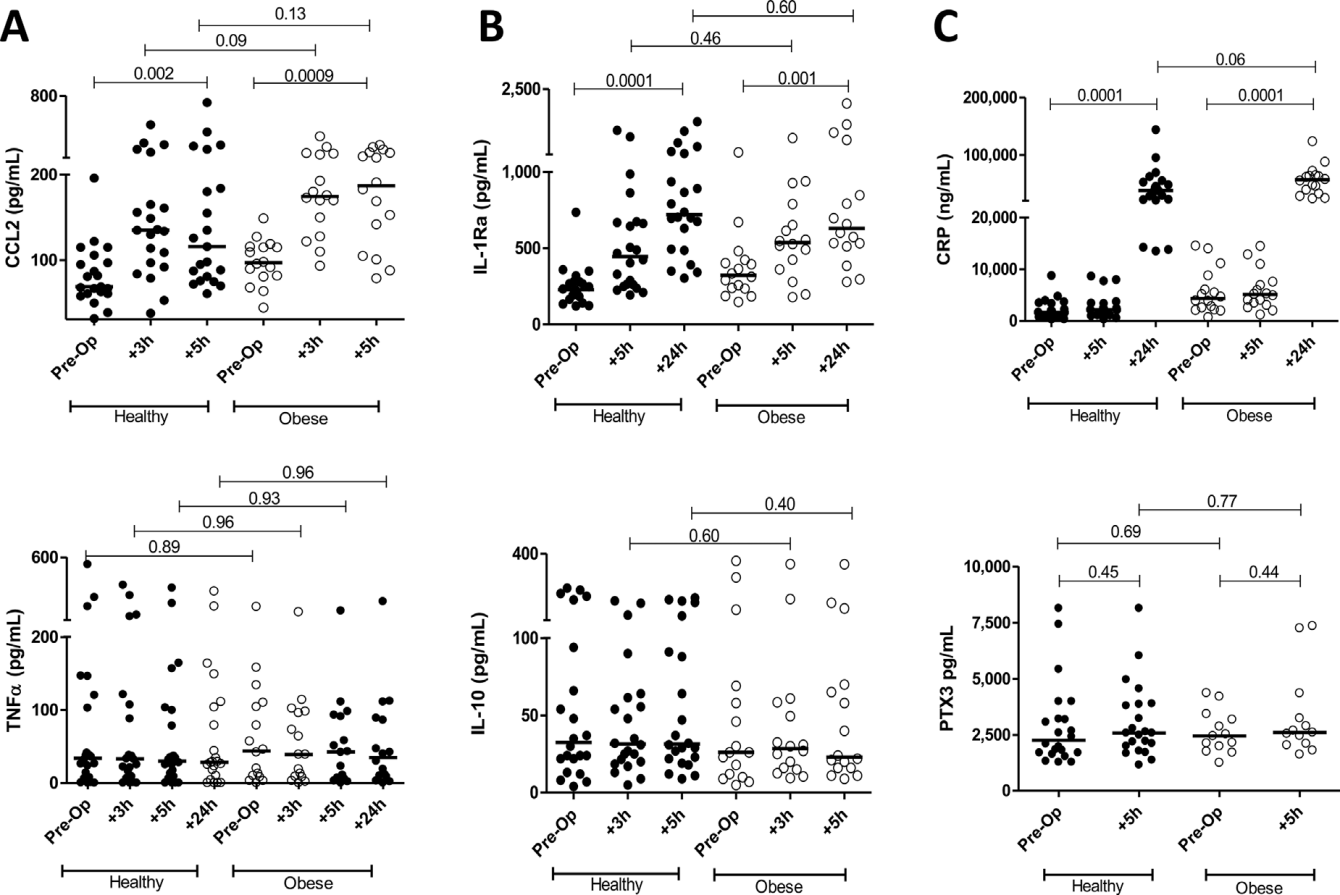
Pro‐ (A) and anti‐(B) inflammatory cytokine expression in response to C‐section in 22 normal weight mothers versus 16 mothers of BMI 35–40 (Class II obesity). (C) Acute phase protein expression. The times presented indicate peak in vivo responses for each biomarker. Wilcoxon matched pairs were used for comparisons within groups versus pre‐operative levels. Mann–Whitney *p*‐values are provided for inter‐group comparisons.

Two hypotheses were tested. Given the chronic mild inflammatory status characteristic of obesity, we hypothesized that obese women undergoing C‐section would exhibit more rapid kinetics of response. However, no differences were evident in the kinetics of pro‐inflammatory, anti‐inflammatory, or acute phase protein responses (Fig. 4). Note that the specific times shown for each cytokine differ slightly so as to capture the development and peak expression for each.

Secondly, the hypothesis that pregnant obese women exhibit more intense innate responses to C‐section surgery than do non‐obese women was tested. Again, systemic responses to surgery for panels of pro‐inflammatory (Fig. 3A), anti‐inflammatory (Fig. 3B), and acute phase proteins (Fig. 3C) were intense yet indistinguishable for each of the biomarkers examined. Thus, neither the kinetics nor intensity of pro/anti inflammatory responses was altered in pregnant women with Class II obesity.

### Innate immune activation to acute PRR‐selective activation in vitro

**Figure 5:**
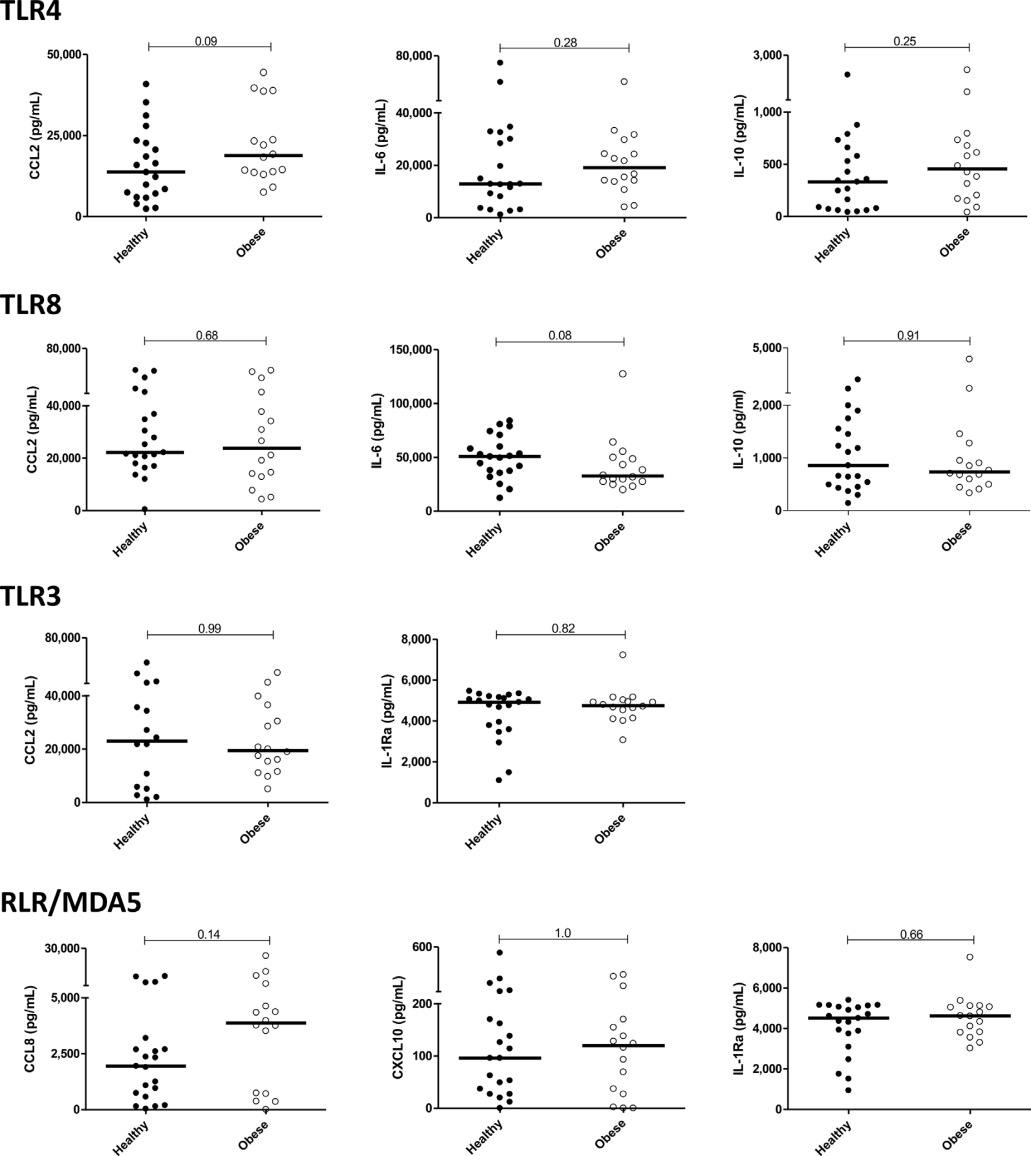
Pro‐ and anti‐inflammatory responses following acute PBMC stimulation with bacterial (TLR4) and viral (TLR8, TLR3, and RLR) PRR ligands. Unstimulated cultures exhibited minimal cytokine production (not shown). Mann–Whitney *p*‐values are shown.

To determine if acute innate immune activation with different stimuli (e.g., PRR‐ligands vs. surgical trauma) elicits differential responses post‐operatively, fresh PBMC were obtained 24 h post‐C‐section and stimulated in vitro with ligands characteristic of bacterial and viral stimuli (Fig. 5). Fresh PBMC were used, rather than purified monocytes, for two reasons. Firstly, an extensive range of circulating cell types (the frequency of each of which varies widely between different individuals) produce cytokines and chemokines upon PRR‐stimulation. We wished to sample the broader repertoire of circulating cells capable of responding to PRR ligation, thereby providing a global view of changes among obese women. Secondly, less intellectually important but logistically relevant, the limited blood volumes available from pregnant women would have reduced the *n* of women available for the study as well as the number of analyses that could be performed with isolated monocytes.

As anticipated, TLR4/LPS stimulation elicited strong pro‐ and anti‐inflammatory responses. The intensity of each response was indistinguishable when comparing normal and obese populations. Similarly, among viral surrogates, TLR8 (CL075) and TLR3 (poly(I:C)), stimulation yielded strong pro‐ and anti‐inflammatory cytokine production in vitro. Again, the two populations of women responded indistinguishably. Finally, examination of a RLR‐stimulus also elicited strong—and indistinguishable—responses in the two populations. Thus, a broad panel of pro‐ and anti‐inflammatory responses evoked by different microbial mimics were vigorous, yet very similar in both populations following elective C‐section.

## Discussion

Obesity is associated with a plethora of undesirable consequences. Despite a wide variety of public health initiatives, it is has been extremely resistant to change 24. Given that C‐section is increasingly common in the developed world, and that obese women are more likely to require C‐section birth 6, improved understanding of complications resulting from such surgery is needed. Here we demonstrate that while pregnant class II obese women (BMI 35–40) exhibit constitutively elevated expression of inflammatory biomarkers (meta‐inflammation) at full term pregnancy, their in vivo and in vitro innate responses to surgery or to innate immune (PRR‐dependent) stimulation are essentially indistinguishable from those of healthy controls.

There is no question that obesity is an undesirable state associated with a multitude of health risks for both mother and, in subsequent years, offspring. However, it is important to assess what biological processes and outcomes are demonstrably affected by elevated BMI and which are not. The data in this report suggest that C‐section in itself does not create a differential impact on a panel of 11 in vivo pro‐, anti‐inflammatory, and acute phase protein responses beyond that normally induced by C‐section itself. Complementing this perhaps surprising conclusion, a related study of inflammation in pregnant rodents fed high fat “cafeteria diets” reached similar conclusions 25. A complementary study, focussed on putatively differential development of inflammation in vivo in lean and obese women over the course of pregnancy (i.e., from recruitment up to delivery), concluded that there was no evidence of additive or synergistic effects between adiposity and pregnancy in terms of endogenous maternal levels of pro‐inflammatory markers during gestation 4.

While these findings appear paradoxical, there is an increasing literature indicating that in some circumstances obesity (<Class II) do not harm and, in some specific circumstances, can be beneficial. For example, low levels of obesity are associated with improved survival during septic shock, with the obese patients exhibiting the lowest 28‐day mortality while lean patients (BMI <25 kg/m^2^) experienced the highest mortality (*p* = 0.02) 26.

This study has caveats that are important for its interpretation. BMI is a widely used but imperfect measure of obesity 19, 27. It does not distinguish lean tissue mass from adipose tissue mass and does not reflect adipose tissue distribution. Measures such as visceral adiposity may provide more specific tools for investigating relationships between obesity and biological outcomes. However, such measures were not available to us for populations of term pregnant women. BMI continues to be widely used today because it is readily determined and is considered by many to be the most useful tool available from a broad‐based health policy perspective 28. Secondly, this study focused on obesity at term and its associated risks for women of elevated BMI (35–40) who elect C‐section surgery. The goal was not to address relative levels of weight gain or their impact on maternal physiology over the term of gestation. Finally, we certainly recognize that of the large number of immune‐regulatory cytokines known, there may be other immune modulators that do exhibit a differential response to C‐section delivery in obese women. Nevertheless, among the 11 pro‐inflammatory, anti‐inflammatory, and acute phase proteins examined, there was no evidence of differential responses in vivo or in vitro.

Several avenues of future research are suggested by our findings. The relative proportion of pro‐ and anti‐inflammatory molecules produced by adipose tissue versus innate immune cells is not known. Because the focus was on the systemic impact of an acute innate immune stimulus (here, the surgical impact of C‐section delivery), net plasma biomarker expression was examined, independent of source. Potential differences over the weeks and months post‐delivery were not examined, nor were inflammation levels in offspring. Similarly, work will be needed to define postoperative complications and clinical status during that period and its relationship to inflammatory responses. Certainly among morbidly obese mothers (BMI > 50), a population less commonly represented in society or successfully pregnant women, the incidence of clinical complications is substantially enhanced in the days and weeks post‐delivery 29.

Finally, interpretation of these findings requires attention to the recruitment criteria utilized here. To test the hypothesis that obesity, in itself, is linked to enhanced inflammatory responses to surgical trauma in pregnant women, participants with hypertension, proteinuria, diabetes, or other ongoing chronic inflammatory diseases—conditions over‐represented in obese populations, were excluded. All participants were healthy, pregnant women. The impact of C‐section on women with extensive co‐morbidities was beyond the scope of this study and will require much larger populations and stratification by the different constellations of comorbidities that are overrepresented in obese women.

## Conclusion

C‐section, like any surgery, elicits systemic responses by multiple pro‐ and anti‐inflammatory constituents. Here we demonstrate that neither the kinetics nor the maximal intensity of a broad panel indicators of innate activation differed when comparing healthy normal and Class II obese women's responses to caesarian section. Similarly, acute stimulation of PBMC ex vivo with a panel of PRR ligands representing mimics of bacterial or viral infection elicited indistinguishable pro‐ and anti‐inflammatory responses in vitro. The data suggest that while Class II obesity is not desirable, it has minimal impact on the in vivo inflammatory response, or innate immune capacity, of women undergoing C‐section.

## Authors’ Contributions

All authors participated in study design. CG carried out immunological assays and analyzed the data. KH and CG drafted the paper. All authors provided substantial input into the final manuscript. DF/MT obtained funding from Health Science Foundation (Manitoba) and University of Manitoba, Department of Anesthesia and Perioperative Medicine Academic Oversight Committee. KH utilized funding from the Canada Research Chairs program. We thank Meghan Azad and Allan Becker for critical review of the manuscript.

## Conflict of Interest

None declared.
